# Change in the Content of Immunoproteasomes and Macrophages in Rat Liver At the Induction of Donor-Specific Tolerance

**Published:** 2017

**Authors:** Ya.D. Karpova, V.D. Ustichenko, N.M. Alabedal’karim, A.A. Stepanova, Yu.V. Lyupina, K.I. Boguslavski, G.A. Bozhok, N.P. Sharova

**Affiliations:** N.K. Koltsov Institute of Developmental Biology, Russian Academy of Sciences, Vavilov Str. 26, Moscow, 119334, Russia; Institute of Problems in Cryobiology and Cryomedicine, National Academy of Sciences of Ukraine, Pereyaslavskaya Str. 23, Kharkov, 61016, Ukraine

**Keywords:** immunoproteasomes, donor specific tolerance, liver, Kupffer cells, rats, flow cytofluorimetry

## Abstract

Induction of donor specific tolerance (DST) by the introduction of donor cells into a recipient’s portal vein is one of the approaches used to solve the problem of transplant engraftment. However, the mechanism of DST development remains unclear to this moment. In the present work, we first studied the change in the content of immunoproteasomes and macrophages of the liver at early stages of the development of allospecific portal tolerance in rats by Western blotting and flow cytofluorimetry. On the basis of the data obtained, we can conclude that the induction of DST is an active process characterized by two phases during which the level of the proteasome immune subunits LMP2 and LMP7 in liver mononuclear cells, including Kupffer cells, and the number of Kupffer cells change. The first phase lasts up to 5 days after the beginning of DST induction; the second phase – from 5 to 14 days. In both phases, the level of the subunits LMP2 and LMP7 in the total pool of mononuclear cells and Kupffer cells increases, with maximum values on days 1 and 7. In addition, the total number of Kupffer cells increases in both phases with a shift in several days. The most noticeable changes take place in the second phase. The third day is characterized by a lower content of mononuclear cells expressing immunoproteasomes compared to the control value in native animals. Presumably, at this time point a “window of opportunity” appears for subsequent filling of an empty niche with cells of different subpopulations and, depending on this fact, the development of tolerance or rejection. The results obtained raise the new tasks of finding ways to influence the cellular composition in the liver and the expression of immunoproteasomes on the third day after the beginning of DST induction to block the development of rejection.

## INTRODUCTION

The problem of transplant engraftment remains one of the concerns in transplantology. Transplantation is used in terminal organ failures, when other methods of treatment have proved ineffective. Allogenic transplantation activates the immune response, which leads to transplant rejection. Modern immunosuppressive protocols are not always able to prevent rejection. Therefore, it is necessary to search for other approaches to induce tolerance to transplants in recipients.

The critical role of the liver in the development of transplant tolerance has been known for a long time. Spontaneous liver allotransplant engraftment in recipients mismatched from donors by the major histocompatibility complex (MHC) was revealed for outbred pigs [1], inbred lineages of mice [2], and rats [3]. Combined transplantation of the liver and other organs led to better engraftment than when using single allografts [4-6].

Another key factor involved in the induction of transplant tolerance is the presence of immunocompe tent cells of donor origin within the liver. This is supported by studies showing that allograft tolerance is not induced after depletion of passenger leukocytes from the donor liver [7-9].

The method of inducing donor-specific tolerance (DST) is based on adherence to these two conditions. The induction is performed by transfusion of donor cells (splenocytes, lymphocytes, bone marrow cells) into the liver via the portal vein. This method leads to significant lifespan extension of allografts of the heart [10], kidneys [11], intestine [12], skin [13], pancreatic islets [14], and trachea [15] in experimental models. However, the molecular-cellular mechanisms of induction and maintenance of DST have not been elucidated, although many researchers stress the significant contribution of hepatic macrophages (Kupffer cells) [16, 17].

Multiple forms of immunoproteasomes that contain the immune subunits LMP2, LMP10, and/or LMP7 with proteolytic activities have been regarded as potential candidates for the role of messengers of immune response, which can direct the immune response either toward allograft acceptance or rejection. Immunoproteasomes participate in the formation of antigenic epitopes for the MHC molecules, the regulation of the expression of co-stimulatory molecules on antigen-presenting cells (APCs), and the differentiation of T-lymphocyte subpopulations [18-21].

We have found previously that the proportion of LMP2 and LMP7 immunoproteasome subunits changes in the liver and allografts of ovaries and thyroid after the induction of DST [22, 23]. Allograft engraftment was accompanied by a significant increase in the quantity of liver mononuclear cells expressing the immunoproteasome subunit LMP2 on the 30th day after DST induction.

Previous experimental and clinical studies have shown that induction of DST fails in some cases [24, 25]. Moreover, 7–15% of recipients develop sensitization to donor antigens [26, 27]. Since the definite mechanism of DST induction is not yet known, it is impossible to predict the vector of the immune response as either transplant acceptance or rejection. This decreases the value of this method and restricts its use in clinical transplantology.

It is evident that the immunological events occurring in the liver of a recipient immediately after the administration of donor cells and that are related to the recognition and presentation of antigen can determine the development of tolerance. Therefore, the study of the cascade of cell-mediated reactions and the change in the pool of proteasomes at early stages after the administration of a donor antigen is important for understanding the mechanism underlying DST induction.

The aim of this study was to evaluate the level of immunoproteasomes and the quantity of resident macrophages in a rat liver in the first two weeks after the beginning of DST induction.

## MATERIALS AND METHODS

**Reagents**

DMEM medium, collagenase and DNAse I (all manufactured by Sigma, USA), percoll (Pharmacia, Sweden), saponin (Calbiochem, USA), rabbit pAb to subunit LMP7, mouse mAb to subunit LMP2 (both manufactured by Biomol International, United Kingdom), phycoerythrin-conjugated mouse mAb to macrophages (Anti-Rat Macrophage Marker, eBioscience, USA), mouse mAb to β-actin (Santa Cruz Biotechnology, USA), Alexa 488-labeled anti-rabbit IgG (Invitrogen, USA), phycoerythrin-conjugated anti-mouse IgG (eBioscience, USA) were used in this study.

**Animals**

The experiments were performed using 5- to 6-month-old Wistar and August female rats. The donors were Wistar rats, and the recipients were August rats. All manipulations with the animals were performed in compliance with the European Convention for the Protection of Vertebrate Animals used for Experimental and Other Scientific Purposes (Strasbourg, 1985). For the experiments, the following groups of animals were used: group 1 (*n *= 12) – intact control; group 2 (*n *= 48) – false-operated animals (an intraportal infusion of a physiological solution); and group 3 (*n *= 48) – animals with DST induction (an intraportal administration of splenocytes); group 4 (*n *= 30) – animals with DST disruption (an intraperitoneal injection of gadolinium chloride GdCl3 (1 mg/100 g of body mass) and an intraportal administration of splenocytes after 24 h).

**Isolation of splenocytes and induction of DST**

All the procedures were performed under sterile conditions. Splenocytes were collected from the spleen of the Wistar rats according to a standard protocol [28]. Erythrocytes were removed through a three-time treatment of a cell suspension with a solution containing 154 mM of ammonium chloride, 10 mM sodium bicarbonate, and 0.082 mM ethylenediamine tetraacetic acid (EDTA). The collected cells were washed two times with a DMEM medium. The average vitality of the splenocytes, assessed with trypan blue staining, was around 90%. DST was induced by administration of 1 ml of a sterile physiological solution containing 1 × 10^7^ splenocytes into the hepatic portal vein. The liver was studied on the 1^st^, 3^rd^, 5^th^, 7^th^, 10^th^, and 14th days after induction.

**Isolation of liver mononuclear cells**

The rat liver was perfused via the portal vein with a calcium-free buffer (5 mM EDTA per 0.1 M phosphate buffer saline, pH 7.4) for 5 min. The liver was then extracted and perfused with 0.1 M phosphate buffer saline containing 0.4 mg/ml collagenase, 3.7 M CaCl_2_, 25 ng/ml DNAse I, and 5 mM MgCl_2_ (pH 7.4) at 37°C for 10 min. Afterwards, the tissue was grinded with scissors and further incubated in collagenase buffer at 37°C for 30 min, disintegrated through pipetting, filtered through a nylon sieve, and centrifuged at 20 *g *at 4°C for 2 min. The supernatant was collected and centrifuged at 400 *g *for 3 min. The pellet of cells was resuspended in 30% percoll and centrifuged at 400 *g *for 30 min at 4°C. The cells were collected from the interface and washed two times with 0.1 M phosphate buffer saline at 4°C.

**Phenotypical analysis of the cells using flow cytofluorimetry**

In order to identify subunits of immunoproteasomes, the isolated hepatic mononuclear cells were fixated in 4% paraformaldehyde for 15 min and permeabilized for 15 min in a 1% saponin solution prepared with 0.1 M phosphate buffer saline. The cells were treated with rabbit pAb to subunit LMP7 and mouse mAb to subunit LMP2 overnight at 4°C in a sample containing 1 × 10^6^ cells and the corresponding antibodies (dilution 1 : 600 per 0.1 M phosphate buffer saline with 1% bovine serum albumin). After washing, the cells were incubated with secondary antibodies: Alexa 488-labelled anti-rabbit IgG or phycoerythrin-conjugated antibodies to mouse IgG at a dilution of 1 : 500 for 30 min at room temperature.

For the identification of Kupffer cells, 1 × 10^6^ cells were resuspended in 0.25 ml of phosphate buffer saline containing 10% fetal bovine serum and incubated for 30 min with phycoerythrin-conjugated anti-mouse mAb (Anti-Rat Macrophage Marker, dilution 1 : 50).

The cells were analyzed using the BD FACSCalibur flow cytometer (BD Bioscience, USA) and the CellQuestPro software.

**Western blotting**

The relative contents of proteasome subunits and β-actin were evaluated in clarified liver homogenates using mouse mAb to subunit LMP7, subunit LMP2, and β-actin as described previously [23].

**Statistical analysis**

Statistical analysis was performed using the Excel and Statistica 7.0 software packages. The data are given as a median; the difference significance between the samples was estimated using a nonparametric Manne– Whitney test with a significance level of 0.05. A Bonferroni correction was used for multiple comparisons.

## RESULTS AND DISCUSSION

**Content of the immunoproteasome subunits LMP2 and LMP7 in cells of the rat liver after intraportal infusion of splenocytes**

**Fig. 1:**
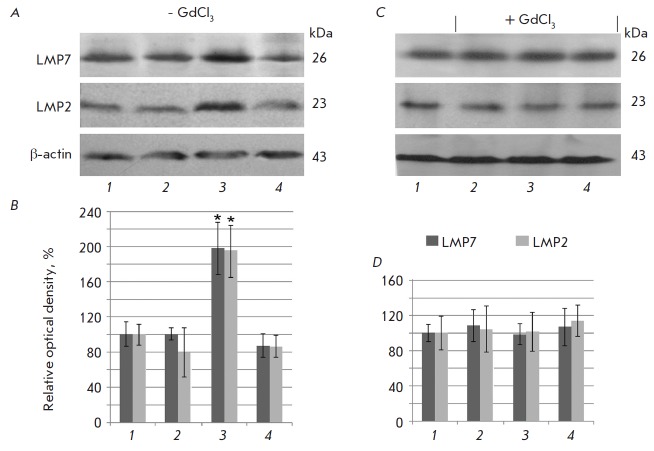
Content of the proteasome subunits LMP7 and LMP2 in clarified homogenates of false-operated rat liver on the 7^th^ day after the introduction of a physiological solution (1) and in rat liver on the 1^st^ day (2), 7^th^ day (3), and 14^th^ day after the beginning of DST induction (4) with preliminary injection of GdCl3 and without it. A, C – Western blots of subunits LMP7, LMP2 and β actin. B, D – Relative quantity (optical density of blots) of subunits LMP7 and LMP2 normalized to β actin content. The subunit quantity in the samples of false-operated animals was taken as 100%; means ± SEM are shown; significant difference at p < 0.05 and n = 5–6 in comparison with the false-operated control is indicated (*).

A Western-blot analysis revealed an elevated level of proteasome subunits in the clarified liver homogenates of five of the six rats with induction of DST (group 3) on the 7^th^ day after the beginning of DST induction compared to the false-operated controls (*Fig. 1*). It is evident that the mechanisms that facilitate the DST effect were impaired in one animal. In the false-operated animals there were no differences in the content of immune subunits at all studied stages after the administration of the physiological solution. No differences were also revealed in the content of immune subunits in the animals of the 4th group with gadolinium chloride infusion. Hepatic mononuclear cells were studied using flow cytofluorimetry in order to establish whether these changes in the immunoproteasome pool are associated with hepatic mononuclear cells.

**Fig. 2:**
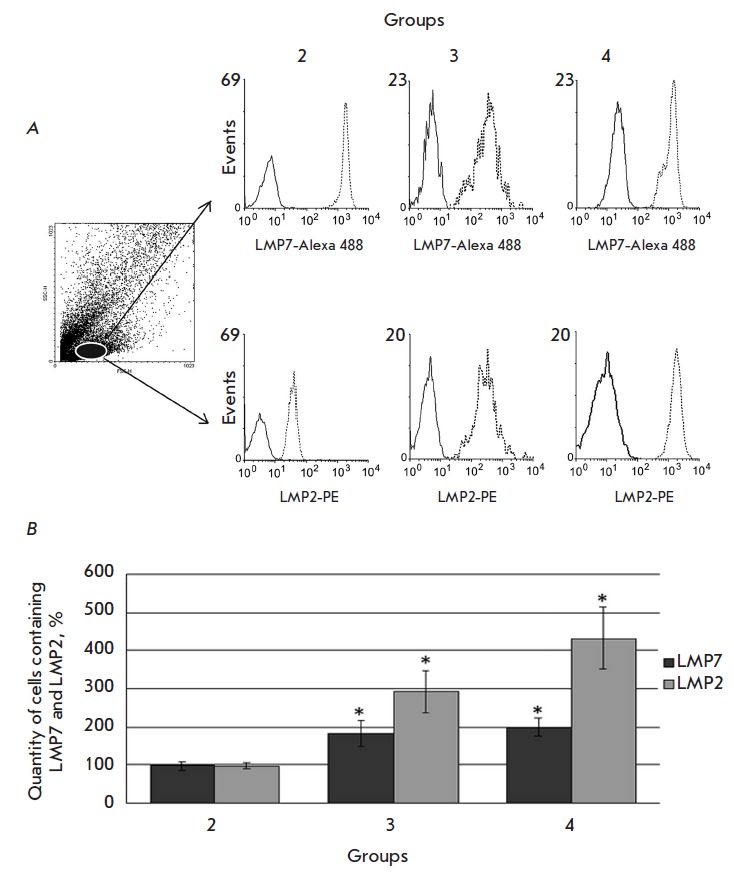
Cytofluorimetric analysis of the expression of the LMP7 and LMP2 subunits in rat liver mononuclear cells. A – In the dotted graph of forward (FSC) and side (SSC) light scattering, the subpopulation of analyzed cells is highlighted by oval. In the right part, the histograms of LMP7 and LMP2 expression in the analyzed cell subpopulation of the liver of the 2^md^, 3^rd^ and 4^th^ rat groups on the 1^st^ day after the beginning of DST induction are presented. B – Per cent of mononuclear cells expressing the LMP2 and LMP7 subunits in the liver of the 2^md^, 3^rd^ and 4^th^ rat groups on the 1^st^ day. In histograms: solid line – isotypical control, dashed line – experiment. The quantity of cells containing the LMP2 and LMP7 subunits in samples of the 1^st^ group was taken as 100%. Significant difference at p < 0.05 and n = 5–6 in comparison with the 2^md^ group is indicated (*).

*Fig. 2A* shows histograms derived during the analysis of the rat hepatic mononuclear cells stained with antibodies to the immune subunits LMP2 and LMP7. The quantity of cells expressing the subunits LMP2 and LMP7 after the beginning of DST induction was found to change (*Fig. 2B*). It was established that as early as on the 1^st^ day, the quantity of LMP7-positive cells increased 1.8 times in the rat livers of both groups with intraportal injection of splenocytes (groups 3 and 4) and the content of LMP2-postivie increased by 3 times compared to the false-operated controls (group 2).

Gadolinium chloride is a widely used specific inhibitor of the antigen presenting the function of Kupffer cells [29]. As shown previously, introduction of this compound to experimental animals 24 h before intraportal infusion of splenocytes abrogates the DST induction phenomenon [13, 16].

We established that the quantity of cells containing the subunits LMP2 and LMP7 in the animals treated with GdCl3 (group 4) did not differ significantly on the 1^st^ day from the quantity of cells in the animals of the 3^rd^ group (without injection of GdCl3), but it was elevated compared to the false-operated animals (group 2) (*Fig. 2B*).

Taking into account the rich composition of hepatic APCs, which, in addition to Kupffer cells and a liver sinusoidal endothelial cell (LSEC), includes dendritic and stellate cells [30], it is logic to conclude that an increase in the number of cells expressing the immune subunits LMP2 and LMP7 can also occur during an inhibition of the macrophage function. However, in this case the quantity of cells containing LMP2 and LMP7 should differ between the 3^rd^ and 4^th^ groups: group 4 should contain fewer of these cells than group 3. The absence of significant differences indicates that the increase in the quantity of cells expressing LMP2 and LMP7 on the 1^st^ day was mostly due to a transfer of donor splenocytes containing immune proteasomes into the liver. Macrophages, even if they contribute to the total number of mononuclear cells enriched in immunoproteasomes in this period, do so in minimal fashion and the contribution does not influence the outcome.

**Fig. 3:**
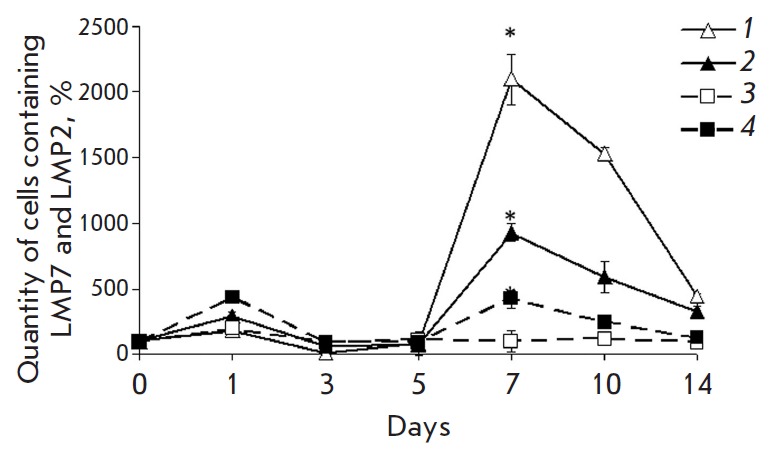
Change in the quantity of mononuclear cells containing the proteasome LMP2 subunit (filled symbols) and LMP7 subunit (empty symbols) in different time intervals after the beginning of DST induction in the liver of the 3^rd^ (lines 1 and 2) and 4^th^ rat groups (lines 3 and 4). On x axis, days after the beginning of DST induction are shown. The quantity of cells containing the LMP2 and LMP7 subunits in samples of the 1^st^ group was taken as 100%. Significant difference at p < 0.05, n = 5 in comparison with basal level (group 1) (*).

The quantity of mononuclear cells containing immunoproteasomes in the liver of the animals of the 3^rd^ group decreased on the 3^rd^ day compared to the 1^st^ day (*Fig. 3*). This could be a result of donor splenocytes leaving the liver of a recipient and migrating to regional lymph nodes [31]. It is also possible that they were eliminated as a result of the activation of recipient cytotoxic CD8^+^ T-lymphocytes [32].

Interestingly, the quantity of mononuclear cells containing immune proteasomes decreases on the 3^rd^ day not only compared to the 1^st^ day of DST induction, but also relative to their basal level in the control animals of the 1^st^ group. Taking into account the fact that immunoproteasomes are expressed mainly in APCs and immunocompetent cells, this fact indirectly points to a decrease in their quantity in the liver on the 3^rd^ day after the beginning of induction. This can be associated with the apoptosis of activated T-lymphocytes observed in the liver during the initiation and maintenance of the tolerance status [31]. However, regardless of the mechanisms involved, the quantity of mononuclear cells enriched in immunoproteasomes in the liver is minimal at this time point, which creates a kind of “window of opportunity” for the subsequent filling of an empty niche with cells of different subpopulations and, depending on this, the development of allospecific tolerance or rejection.

On the 7^th^ day after the infusion of splenocytes, the maximum rise in the content of mononuclear cells expressing immunoproteasomes was observed in the liver of animals of the 3^rd^ group, which exceeded the values for the control group almost 100 times for LMP2, and 200 times for LMP7 (*Fig. 3*). This excellent response could be rooted in both the transfer of immunocompetent cells into the liver in response to the infusion of donor splenocytes and the activation of the resident APC pool in the liver itself, which is accompanied by an increase in the content of immune subunits [33, 34]. In the subsequent days, the quantity of cells expressing immune subunits gradually decreased.

On the whole, the results of flow cytofluorimetry are consistent with Western blot findings that indicate a burst in immunoproteasome expression in the liver on the 7^th^ day after DST induction. In addition, the discovery of this effect not in all the animals supports the hypothesis of the different possibilities of niche filling after the 3^rd^ day that is critical for the development of tolerance or rejection.

In animals treated with GdCl3, there was no similar noticeable increase in the amount of cells enriched in immunoproteasomes. The quantity of LMP7-positive cells did not differ from their quantity in false-operated animals, and the number of LMP2-positive cells on the 7^th^ day exceeded the control values only four times. The difference between groups 3 and 4 indirectly indicates that the inhibition of Kupffer cells influences the processes dependent on the immunoproteasomes that occur in the early stages of DST induction.

**Relationship between the content of Kupffer cells and the change in the expression of immunoproteasomes during DST induction**

Our results led to a need for a direct assessment of the Kupffer cell content in different periods after splenocyte infusion. We used monoclonal antibodies that recognize ED2-like antigens on the membranes of rat resident macrophages, including Kupffer cells [35].

**Fig. 4:**
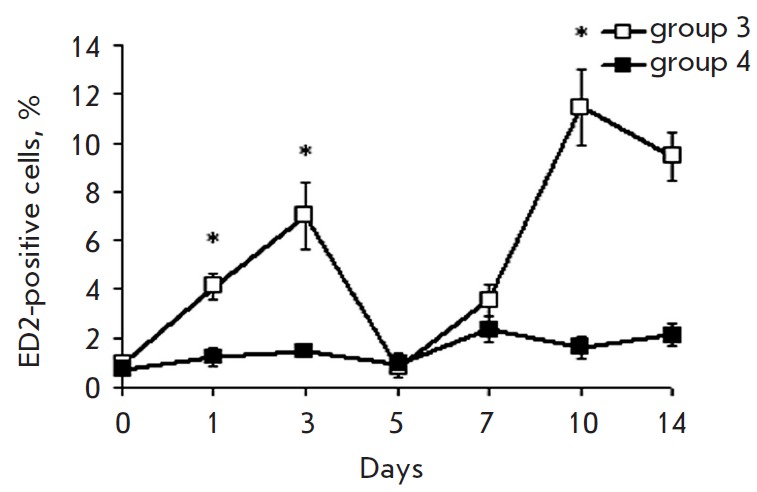
Cytofluorimetric analysis of cells expressing the macrophage ED marker in the 3^rd^ and 4^th^ rat groups at different stages after introduction of donor splenocytes. On x axis, days after the beginning of DST induction are shown. The quantity of cells in samples of the 1^st^ group was taken as 100%. Significant difference at p < 0.05, n = 5 in comparison with basal level (group 1) (*).

The profile of the dynamics of ED2-positive cells also had two maximums (*Fig. 4*), with the first peak occurring with a two-day shift and the second peak with a three-day shift later compared to the peaks in the content of the total mononuclear cell pool expressing immunoproteasomes (*Fig. 3* and *Fig. 4*). This shift can be accounted for by the fact that, at first, APCs present a foreign alloantigen with the participation of immunoproteasomes. This process is accompanied by a release of mediators of the immune response, which serve as a signal for the proliferation of Kupffer cells [36, 37].

In animals of the 4^th^ group, there were no bursts in the quantity of macrophages in the liver, probably because of the absence or defect stage of antigen presentation after the infusion of GdCl_3_.

The results make it possible to state that induction of portal tolerance is an active process that affects several subpopulations of liver APCs and makes it possible for the rearrangements in the intracellular proteasome pool to be involved in the mechanisms of antigen processing and presentation. In addition, in the early stages of DST development, two waves were observed: the first (1–3 days) was associated with the transfer of donor immune system cells into the liver; the second (7–10 days) – with the activation of a response in the liver of a recipient involving Kupffer cells.

Does the expression profile of the inducible subunits LMP2 and LMP7 change in Kupffer cells after intraportal alloantigen infusion?

**Fig. 5:**
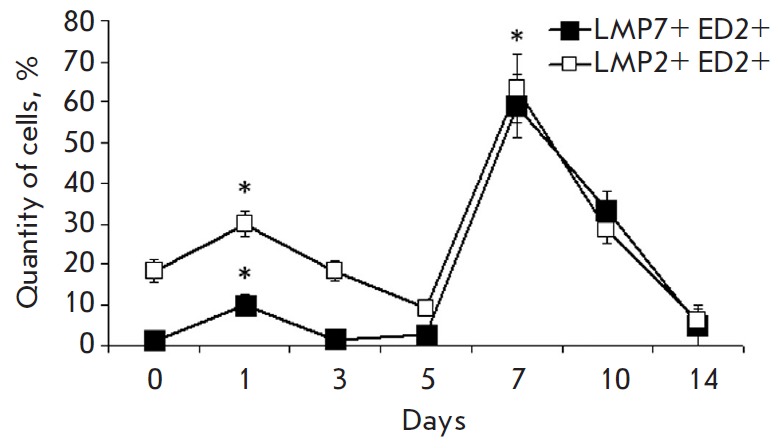
Cytofluorimetric analysis of LMP7 and LMP2 subunit expression in ED2-positive cells of the liver of the 3^rd^ rat group after the beginning of DST induction. On x axis, days after the beginning of DST induction are shown. The total quantity of ED2-positive cells was taken as 100%. Significant difference at p < 0.05, n = 5 in comparison with basal level (group 1) (*).

In order to answer this question, we studied the changes that occurred in the proteasome pool of ED2- positive cells in rat liver with DST induction (group 3) in different periods after the administration of donor splenocytes (*Fig. 5*). First, two peaks of an increase in the quantity of the subunits LMP2 and LMP7 – on the 1^st^ and 7^th^ days – were revealed. Second, it was revealed that the proportion of LMP2 and LMP7 subunit expression in ED2-positive cells changes with time after DST induction. During the first 5 days, the quantity of LMP2 increased more noticeably than that of LMP7; the level of both immune subunits was similarly high on the 7^th^ day.

The time changes in the content of ED2-positive cells and immunoproteasomes were characterized by two phases. In the first phase, the quantity of LMP2 and LMP7 increased on the 1^st^ day, similar to the one in the total pool of mononuclear cells, while the quantity of ED2-positive cells increased on the 3^rd^ day (*Fig. 3*, *Fig. 4*, *Fig. 5*). In the second phase, all these events occurred in the same sequence: a peak in the expression of immunoproteasome subunits in ED2-positive cells was observed on the 7^th^ day, similar to that in the total mononuclear cell pool, followed by an increase in the quantity of ED2- positive cells on the 10^th^ day.

It is likely that differences exist in the mechanisms underlying the increase in the expression of immunoproteasome subunits in the total mononuclear cell pool in the first and second phases. The first peak of expression of LMP2 and LMP7 subunits reflects mostly the transfer of splenocytes enriched in immunoproteasomes. At the same time, to a greater or a lesser extent the second peak can be associated with *de novo *LMP2 and LMP7 subunit synthesis in mononuclear cells of a recipient’s liver, including in Kupffer cells. This synthesis is induced in the first phase, resulting from the encounter of splenocytes with hepatic APCs. LSEC are known to cross-present a foreign antigen immediately to CD8^+^ T-lymphocytes [38], and the process requires an insignificant quantity of stimulatory biomaterial (< 1 nM). This process takes several hours [39] and is accompanied by the release of cytokines [40, 41] that send signals for expression upregulation of the inducible subunits LMP7 and LMP2 [21, 42]. The hypothesis of a *de novo *immunoproteasome synthesis in the second phase is also supported by the fact that the expression of immunoproteasome subunits in response to cytokines reaches a peak only in 5–7 days [43, 44]. The first peak of an increase in the level of proteasome subunits in Kupffer cells can reflect the initial stage of their *de novo *synthesis.

The proportion of proteasome immune subunits influences the activation of macrophages and their polarization into either an ED1- or ED2-phenotype [45]. Therefore, the change in LMP2 and LMP7 subunit levels in cell subpopulations can be associated with the activation of macrophages type 2. This in turn explains the prevalence of processes in the liver that are involved in the prevention of rejection reactions due to the fact that ED2-macrophages belong to the anti-inflammatory functional phenotype, which is characterized by the secretion of the cytokines IL-10, IL-4, and TGF-β [46].

The established dynamics of immunoproteasome expression in hepatic mononuclear cells reflects the changes in the reactivity of their subpopulations in response to the introduction of foreign antigens. Previous data showed the appearance of peaks reflecting cell activation within the liver after peptide antigen administration or adoptive transfer of lymphocytes. For example, the proliferation of donor cells occurred on the 2.5^th^ and 6th days after adoptive transfer of CD8^+^ T-lymphocytes in the liver of a recipient [47]. An eight-fold expansion of the CD8^+^ T-lymphocyte subpopulation on the 2^md^ day after their intraportal infusion, followed by a gradual decline by the 4^th^ day, was also revealed [48]. Stimulation with antigenic peptide SEFLLEKRI led to a 100-fold expansion of the mononuclear cell pool within the liver, starting from the 2^md^ day, followed by response extinction by the 6th day [49]. It is interesting to note that the lymphocyte proliferation peaked on the 4^th^ day and the dynamics of the rest subpopulations was biphasic, with peaks on the 1^st^ and 4^th^ days.

**Fig. 6:**
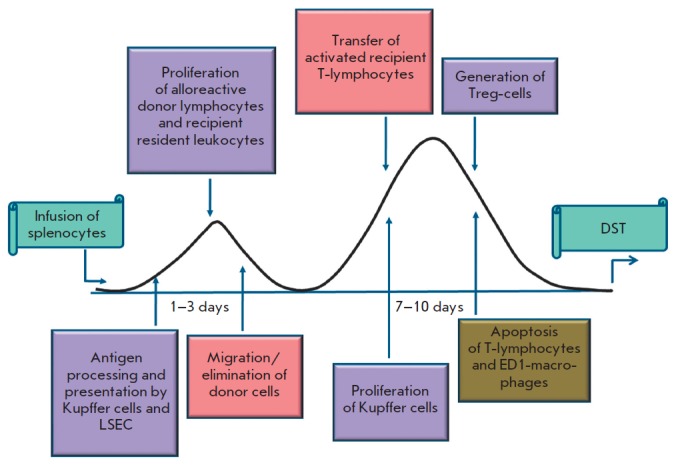
Scheme of DST induction and development.

Thus, a strong immunologic basis underlies the biphasic pattern of immune reactivity of the liver in response to intraportal infusion of a donor antigen (*Fig. 6*). In the first phase, LSEC and Kupffer cells encounter donor cells, which, due to the specific ability of the liver to retain activated CD8^+^ T-lymphocytes [48], remain there for a sufficient time for antigen presentation. After processing and antigen presentation with the involvement of immunoproteasomes, the proliferation of donor leukocytes and resident hepatic immunocompetent cells is triggered. Antigen presentation and activation of lymphocytes are accompanied by the release of cytokines, which play a leading role in the recruiting of the macrophages and lymphocytes of a recipient into the liver [50, 51]. This results in the appearance of the second peak in the dynamics of the hepatic immunoproteasome pool after DST induction.

The interaction of activated CD8^+^ T-lymphocytes with Kupffer cells leads to their apoptosis due to the absence of adequate proinflammatory stimulation [48]. In addition to the direct interaction, Kupffer cells produce some proapoptotic substances, such as TNF-α, CD95-ligand, galectin-1, and indoleamine-dioxygenase [52, 53]. A reduction in lymphocyte quantity at the end of the first phase during DST induction leads to a decline in the hepatic immunoproteasome pool. A further decrease in the quantity of immunoproteasomes in this period can occur due to the migration of donor cells into the blood flow of a recipient.

The ability of immunocompetent hepatic cell subpopulations of primary antigen presentation [51], which results in the deletion of alloreactive CD8^+^ T-lymphocytes via apoptosis, in the absence of positive costimulation [48], presents an opportunity for the prevention of the development of an immune response in the first days (1–3) after the administration of donor cells.

The second phase is associated with the clonal expansion and transfer of the activated T-lymphocytes and macrophages of a recipient into the liver. The phenotypical profile of the cells that fill the immunological niche of the liver in this phase likely contributes to either tolerance or rejection reactions. Activation of immune defense mechanisms in the liver directed towards eliminating/alleviating inflammation can shift the balance in the side of tolerance. These mechanisms include the apoptosis of the activated T-lymphocytes of a recipient [31], ED1-polarized macrophages and activated T-cells in the presence of ED2-phenotype macrophages in the liver [54, 55], and the expansion of Treg-cells [56] in response to antigen presentation in the liver.

The repertoire of immune subunits influences the hierarchy of the presented antigenic epitopes on APCs. At least four forms of immune proteasomes have been described. One form contains all three proteolytic immune subunits: LMP7, LMP2, and LMP10. Two forms contain two immune subunits and one proteolytic constitutive subunit: β5-LMP2-LMP10 and LMP7- LMP2-β2. One form contains the immune subunit LMP7 and two constitutive subunits β1 and β2 [57-60]. The combination of subunits with proteolytic activity determines the changes in the conformation of substrate-binding pockets [61], the preferable sites for protein hydrolysis and, therefore, the range of produced antigenic epitopes. Hence, the change in the balance of immune subunits in resident and transitory immune system cell subpopulations plays an important role in how a foreign antigen is presented, either as a “non-self” molecule to cause rejection or as a “self” molecule to be accepted.

## CONCLUSIONS

This paper has studied for the first time the changes that occur in the immunoproteasome pool of mononuclear cells within the liver during the induction of allospecific portal tolerance. Based on the findings, it is concluded that DST induction is an active process characterized by two phases wherein the proportion of the immunoproteasome subunits LMP2 and LMP7 and the quantity of hepatic APCs, including Kupffer cells, change. Apparently, the balance of these parameters is important for the development of tolerance to transplanted tissues. The third day after the beginning of DST induction is the key point when a kind of “window of opportunity” opens for a subsequent filling of an empty niche with cells of different subpopulations and, depending on this factor, the development of either tolerance or rejection. The results present new tasks related to the search for ways to influence the cellular composition of the liver and the expression of immunoproteasomes on the 3^rd^ day after the beginning of DST induction for blocking rejection.

## Abbreviations

MHC: major histocompatibility complex
DST: donor specific tolerance
APC: antigen presenting cells
LSEC: liver sinusoid endothelial cells
Ab: antibodies
pAb: polyclonal antibodies
mAb: monoclonal antibodies

## References

[1] Calne R.Y., Sells R.A., Pena J.R ., Davis D. R., Millard P.R., Herbertson B.M., Binns R.M., Davies D.A. (1969). Nature. 1969. V. 2. № 223 (5205)..

[2] Qian S., Demetris A., Murase N., Rao A.S., Fung J.J., Starzi T.E. (1994). Hepatology.

[3] Zimmermann F.A., Davies H.S., Knoll P.P. (1984). Transplantation..

[4] Kamada N., Wight D.G.D. (1984). Transplantation..

[5] Cunningham E.C., Sharland A.F., Bishop G.A. (2013). Clin. Dev. Immunol..

[6] Topilsky Y., Raichlin E., Hasin T., Boilson B.A., Schriger J.A., Pereira N.L., Edwards B.C., Schriger J.A., Pereira N.L., Edwards B.S., Topilsky Y., Raichlin E., Hasin T. (2013). Transplantation..

[7] Sun J., McCaughan G.W., Gallagher N.D., Scheil A.G., Bishop G.A. (1995). Transplantation..

[8] Shimizu Y., Goto S., Lord R., Vari F., Edwards-Smith C., Chiba S., Schlect D., Buckley M., Kusano M., Kamada N. (1996). Transpl. Int..

[9] Ko S., Deiwick A., Jager M.D., Dinkel A., Rohde F., Fisher R.T., Tsui T.Y., Rittmann K.L., Wonigeit K., Schlitt H.J. (1999). Nat. Med..

[10] Kenick S., Lowry R.P., Forbes R.D.S., Lisbona R. (1987). Transplant. Proc..

[11] Oko A., Idasiak-Piechocka I., Pawlaczyk K., Wruk M., Pawlaczyk E., Czekalski S. (2002). Ann. Transplant..

[12] Sheng Sun D., Iwagaki H., Ozaki M., Ogino T., Kusaka S., Fujimoto Y., Murata H., Sadamori H., Matsukawa H., Tanaka N., Yagi T. (2005). Transpl. Immunol..

[13] Diaz-Peromingo J.A., Gonzalez-Quintela A. (2005). Eur. Surg. Res..

[14] Ikebukuro K., Adachi Y., Yamada Y., Fujimoto S., Seino Y., Oyaizu H., Hioki K., Ikehara S. (2002). Transplantation..

[15] Chalermskulrat W., McKinnon K.P., Brickey.J. W., Neuringer I.P., Park R.C., Sterka D.C., Long B.R., McNeillie P., Noelle R.J., Ting J.P., Aris R.M. (2006). Thorax..

[16] Nakagawa K., Matsuno. T., Iwagaki H., Morimoto Y., Fujiwara T., Sadamori H., Inagaki M., Urushihara N., Yagi T., Tanaka N. (2001). J. Int. Med. Res..

[17] Watanabe T., Kudo M., Chiba T., Wakatsuki Y. (2008). Hepatol. Res..

[18] Gaczynska M., Rock K.L., Goldberg A.L. (1993). Enzyme Protein..

[19] Cong Y., Konrad A., Iqbal N., Hatton R.D., Weaver C.T., Elson C.O. (2005). J. Immunol..

[20] Trombetta E.S., Mellman I. (2005). Annu. Rev. Immunol..

[21] Basler M., Kirk C.J., Groettrup M. (2013). Curr. Opin. Immunol..

[22] Karpova Ya.D., Bozhok G.A., Lyupina Yu.V., Legach E.I., Astakhova T.M., Stepanova A.A., Bondarenko T.P., Sharova N.P. (2012). Izvestiia Rosiiskoi Akademii nauk. Seriia biologicheskaia..

[23] Stepanova A.A., Karpova Y.D., Bozhok G.A., Ustichenko V.D., Lyupina Y.V., Legach E.I., Vagida M.S., Kazansky D.B., Bondarenko T.P., Sharova N.P. (2014). Bioorg. Khim..

[24] Yunusov M.Y., Kuhr C.S., Georges G.E., Hogan W.J., Taranova A.G., Lesnikova M., Kim Y.S., Nash R.A. (2006). Transplantation..

[25] Bozhok G.A. (2011). Problemy endokrynnoi patolohii..

[26] Marti H.P., Henschkowski J., Laux G., Vogt D., Seiler C., Opelz G., Frey F.J. (2006). Transpl. Int..

[27] Mackie F. (2010). Nephrology (Carlton)..

[28] (1990). Lymphocytes: A practical approach. Ed. by G.G.B. Klaus. M.: Mir. 1990. 395 p..

[29] Ahmad N., Gardner C.R., Yurkow E.I., Laskin D.L. (1999). Hepatology..

[30] Crispe I. N., Giannandrea M., Klein I., John B., Sampson B., Wuensch S. (2006). Immunol. Reviews..

[31] Bishop G.A., Wang C., Sharland A.F., McCaughan G. (2002). Immunol. Cell Biol..

[32] Fast L.D. (1996). J. Immunol..

[33] Stevanovic S. (2002). Transpl. Immunol..

[34] Jin Y., Fuller L., Ciancio G., Burke G.W. 3rd., Tzakis A.G., Ricordi C., Miller J., Esquenzal V. (2004). Hum. Immunol..

[35] Polfliet M.M., Fabriek B.O., Daniëls W.P., Dijkstra C.D., van den Berg T.K. (2006). Immunobiology..

[36] Milner J.D., Orekov T., Ward J.M., Torres-Velez F., Junttila I., Sun G., Buller M., Morris S.C., Finkelmann F.D., Paul W.E. (2010). Blood..

[37] Jenkins S.J., Ruckerl D., Cook P.C., Jones L.H., Finkelman F.D., van Rooijen N., MacDonald A.S., Allen J.E. (2011). Science..

[38] Knolle P.A., Limmer A. (2003). Swiss Med. Wkly..

[39] Limmer A., Ohl J., Wingender G., Berg M., Jungerkes F., Schumak B., Djandji D., Scholz K., Klevenz A., Hegenbarth S. (2005). Eur. J. Immunol..

[40] Limmer A., Ohl J., Kurts C., Ljunggrens H.G., Reiss Y., Groettrup M., Momburg F., Arnold B., Knolle P.A. (2000). Nat. Med..

[41] Schurich A., Berg M., Stabenow D., Bottcher J., Kern M., Schild H.J., Kurts C., Schuette V., Burgdorf S., Dieh lL., Limmer A., Knolle P.A. (2010). J. Immunol..

[42] Niewerth D., Kaspers G.J., Assaraf Y.G., van Meerloo J., Kirk C.J., Anderl J., Blank J.L., van de Ven P.M., Zweegman S., Jansen G. (2014). J. Hematol. Oncol..

[43] Khan S., van den Broek M., Schwarz K., de Giuli R., Diener P.A., Groettrup M. (2001). J. Immunol..

[44] Heink S., Ludwig D., Kloetzel P.M., Kruger E. (2005). Proc. Natl. Acad. Sci. USA..

[45] Chen S., Kammerl I.E., Vosyka O., Baumann T., Yu Y., Wu Y., Irmler M., Overkleeft H.S., Beckers J., Eickelberg O., Meiners S., Stoeger T. (2016). Cell Death Differ..

[46] Tcke F., Zimmermann H.W. (2014). J. Hepatol..

[47] Bowen D.G., Zen M., Holz L., Davis T., McCaughan G.W., Bertolino P. (2004). J. Clin. Invest..

[48] Kuniyasu Y., Marfani S.M., Inayat I.B., Sheikh S.Z., Mehal W.Z. (2004). Hepatology..

[49] Huang L., Soldevila G., Leeker M., Flavell R., Crispe I.N. (1994). Immunity..

[50] Lalor P.F., Shields P., Grant A., Adams D.H. (2002). Immunol. Cell Biol..

[51] Robinson M.W., Harmon C., O’Farrelly C. (2016). Cell Mol. Immunol..

[52] Perillo N.L., Pace K.E., Seilhamer J.J., Baum L.G. (1995). Nature.

[53] Müschen M., Warskulat U., Peters-Regehr T., Bode J.G., Kubitz R., Häussinger D. (1999). Gastroenterology..

[54] You Q., Cheng L., Kedl R.M., Ju C. (2008). Hepatology..

[55] Wan J., Benkdane M., Teixeira-Clerc F., Bonnafous S., Louvet A., Lafdil F., Pecker F., Tran A., Gual P., Mallat A. (2014). Hepatology..

[56] Dangi A., Sumpter T.L., Kimura S., Stolz D.B., Murase N., Raimondi G., Vodovotz Y., Huang C., Thomson A.W., Gandhi C.R. (2012). J. Immunol..

[57] Groettrup M., Standera S., Stohwasser R., Kloetzel P.M. (1997). Proc. Natl. Acad. Sci. USA..

[58] Griffin T.A., Nandi D., Cruz M., Fehling H.J., van Kaer L., Monaco J.J., Colbert A. (1998). J. Exp. Med..

[59] Guillaume B., Chapiro J., Stroobant V., Colau D., van Holle B., Parvizi G., Bousquet-Dubouch M.P., Théate I., Parmentier N., van den Eynde B.J. (2010). Proc. Natl. Acad. Sci. USA..

[60] Dahlmann B. (2016). Arch. Biochem. Biophys..

[61] Unno M., Mizushima T., Morimoto Yu., Tomisugi Y., Tanaka K., Yasuoka N., Tsukihara T. (2002). Structure..

